# Eligibility for faricimab in a real-world diabetic macular oedema population: a cross-sectional study

**DOI:** 10.1136/bmjopen-2024-089801

**Published:** 2025-02-05

**Authors:** Inger Westborg, Ayat Al-Najjar, Helena Norberg

**Affiliations:** 1 Department of Clinical Sciences/Ophthalmology, Umea University Faculty of Medicine, Umea, Sweden; 2 Department of Medical and Translational Biology, Umea University Faculty of Medicine, Umea, Sweden

**Keywords:** Diabetic retinopathy, OPHTHALMOLOGY, Cross-Sectional Studies, Drug Therapy, REGISTRIES

## Abstract

**Abstract:**

**Purpose:**

To investigate the eligibility for faricimab in a real-world diabetic macular oedema (DMO) population to the YOSEMITE and RHINE trials, and to compare the eligible DMO populations to the trial populations.

**Design, settings and participants:**

This retrospective cross-sectional analysis used data from the Swedish Macula Registry (SMR) between 1 January 2019 and 31 August 2023. Eligibility criteria mirrored the main criteria of the YOSEMITE and RHINE trials: (1) DMO diagnosis, (2) treatment-naïve, (3) 18 years or older, (4) central retinal thickness (CRT) 325 µm or higher and (5) best-corrected visual acuity (BCVA) ranging from 25 to 73 letters. Individuals with registered proliferative diabetic retinopathy (DR) at the start of treatment were excluded. A secondary selection of eligible individuals was conducted using the same criteria, except for BCVA, which ranged from 25 to 77 letters according to national guidelines (treatment practice).

**Main outcome measures:**

Characteristics at the initial visit of the two eligible SMR populations were compared with baseline data from the clinical trials, respectively.

**Results:**

In total, 3777 individuals with DMO were selected from SMR. Of these, 2357 (62.4%) individuals were treatment-naïve, all were 18 years or older, 1928 (51.0%) exhibited CRT≥325 µm, 1175 (31.1%) had 25–73 letters based on phase III studies, while 1528 (40.5%) had 25–77 letters according to treatment practice. After excluding individuals with registered proliferative DR 1171 (31.0%) individuals in the SMR met all criteria based on phase III studies, while 1522 (40.3%) individuals fulfilled the criteria according to treatment practice. The SMR and treatment practice populations were older (YOSEMITE 67.5±11.6 vs 62.8±10.0 years, p<0.001 and RHINE 67.5±11.6 vs 61.6±10.1 years, p<0.001) than those in the phase III studies and had lower CRT (YOSEMITE 446±96 vs 486±131 µm, p<0.001 and RHINE 446±96 vs 471±127 µm, p=0.001).

**Conclusion:**

Approximately 30% of DMO patients in the SMR met the main trial criteria from YOSEMITE and RHINE, while around 40% met the criteria based on treatment practice. The SMR and treatment practice cohorts were older and had less severe DMO than the trial cohorts. Further research into the safety profile of faricimab in clinical settings is necessary, along with the consideration of additional eligibility criteria when implementing faricimab in ophthalmology practice.

**STRENGTHS AND LIMITATIONS OF THIS STUDY**

- This study investigated a nationwide, real-world diabetic macular oedema population spanning 4.7 years.
- The Swedish Macula Registry (SMR), national quality register with a coverage of 75%–80% of all clinics in Sweden.
- Limitations of this study include incomplete data for some of the variables because not all variables are mandatory in the SMR.
- A more pragmatic approach was adopted compared with the entry criteria in the YOSEMITE and RHINE trials, which impacts the results.

## Introduction

 Diabetic retinopathy (DR) ranks among the primary contributors to preventable vision loss and blindness worldwide.^1^ DR is characterised by damage to the blood vessels in the retina, often leading to diabetic macular oedema (DMO).^2^ The global prevalence of DMO is approximately 5.5% in individuals with diabetes.^3^ DMO poses a significant threat to vision and occurs more frequently with more severe stages of DR.^4^ As the number of people with diabetes is expected to increase over time, it becomes evident that the prevalence of DMO will also rise.^5 6^

Prolonged diabetes duration, elevated systolic blood pressure and increased haemoglobin A1C levels enhance the risk of DMO.^4^ DMO involves fluid accumulation in the macula due to leakage from hyperpermeable retinal capillaries, exacerbated by elevated vascular endothelial growth factor (VEGF) levels resulting from prolonged hyperglycaemia. This impairs the eye’s ability to clear serum, leading to macular swelling and potential vision impairment.

In recent years, the treatment landscape for DMO has evolved significantly. Localised laser therapy, once the standard approach, has been replaced by anti-VEGF agents as first-line treatment.^7^ Corticosteroids and occasionally surgery serve as alternative options. However, corticosteroids carry the risk of increased intraocular pressure and accelerated cataract development, relegating them to secondary treatment choices. Hence, there remains interest in developing new treatments with improved efficacy, longer duration and enhanced cost-effectiveness.

Faricimab is a novel, humanised, bispecific antibody that received regulatory approval from the US Food and Drug Administration, and the European Medicines Agency in 2022.^8^ Faricimab inhibits the activity of VEGF-A and angiopoietin-2 and is suitable for the treatment of neovascular age-related macular degeneration (nAMD) and DMO. The phase III trials YOSEMITE and RHINE demonstrated that DMO treatment with faricimab, with treatment intervals of up to 16 weeks, was non-inferior in terms of vascular stability and reduced vascular leakage, and exhibited potential anti-inflammatory effects compared with aflibercept 2 mg administered every 8 weeks.^9^

Faricimab’s utilisation in Sweden is so far limited due to ongoing regional hospital procurement procedures, resulting in a lack of substantial real-world data regarding its effectiveness and safety. To address this gap, the seven-step systematic implementation approach can be a valuable tool.^10^ By employing this approach, healthcare providers can systematically identify individuals best suited for novel medications like faricimab. This approach enhances implementation control, enabling resources to be strategically allocated to individuals with the highest likelihood of deriving therapeutic benefits.

The aim of this study was to examine the eligibility for faricimab and to assess how applicable the findings from clinical trials are to real-world patients with DMO registered in the Swedish Macula Registry (SMR) by employing the first two steps of the seven-step systematic implementation approach. The study seeks to answer the following questions: (1) what percentage of real-world DMO populations would meet the main criteria for receiving faricimab as outlined in the YOSEMITE and RHINE trials? (2) how similar are the populations in the YOSEMITE and RHINE trials to those eligible in real-world scenarios?

## Methods

### Population and register

Data for this cross-sectional study were obtained from the SMR spanning from 1 January 2019 to 31 August 2023. SMR is a national quality registry established in 2003, transitioning to a web-based platform in 2008.^11^ Originally founded for the treatment of nAMD, SMR expanded its scope in response to requests to include other eye conditions treated with anti-VEGF medications, such as DMO, which was incorporated into the registry in 2019. The registry encompasses various variables including diagnosis, age, gender, type of treatment, adverse events, visual acuity, among others. Coverage analysis for DMO within SMR has not yet been conducted, yet participation rates are high, with 80% of Swedish eye clinics involved as of 2023.

Individuals voluntarily enrolled in the registry were informed about the use of their data for quality assessments, statistics and research purposes after ethical approval, and that they have the right to decline registration or opt out later.

### Selection process and eligibility criteria

This study applied a previously published systematic approach for the local implementation of novel therapies in individuals with chronic diseases in Sweden.^10^ The model, consisting of seven steps, has previously been used to introduce new heart failure and anti-VEGF medications into real-world clinical practice and is adaptable to various disciplines.^12^,^14^ It is advisable to complete the initial two steps prior to the marketing approval for a medication, as this allows for a more accurate estimation of the proportion of eligible individuals in real-world settings.^10^ Given that faricimab is a novel medication for DMO soon to be introduced in Sweden, steps 1 and 2 of the implementation model were employed. Step 1 involves defining specific treatment criteria, while step 2 entails conducting a primary scan using registries, databases or medical records to identify eligible individuals based on these criteria.

During step 1, consultations were conducted with an ophthalmologist to deliberate on suitable criteria drawn from the phase III trials of YOSEMITE and RHINE.^9^ Following consensus, five clinically relevant inclusion criteria and one exclusion criterion were selected: (1) DMO diagnosis, (2) treatment-naïve, (3) 18 years or older, (4) central retinal thickness (CRT) 325 µm or higher (defined as the average thickness between the internal limiting membrane and Bruch’s membrane in the central 1 mm diameter of the Early Treatment Diabetic Retinopathy Study (ETDRS) grid) and (5) best-corrected visual acuity (BCVA) ranging from 25 to 73 ETDRS letters (otherwise, approximative ETDRS calculated on Snellen^15^). Individuals with registered proliferative DR at the initiation of DMO treatment were excluded as it could be an incorrect indication registered.

Additionally, secondary screening of eligible individuals was performed using the same criteria, except for BCVA, which ranged from 25 to 77 ETDRS letters. This was due to the national guidelines and we wanted to study the results from this perspective as it could have implications on initiation and treatment practice in Sweden.^16^

Regarding criterion two, YOSEMITE and RHINE stipulated that individuals should either be treatment-naïve or have received treatment previously, provided that the treatment occurred no later than 3 months before the start of the study.^9^ Hence, YOSEMITE and RHINE allowed for 22% and 20% non-treatment-naïve individuals. Because SMR lacks information regarding the timing of prior treatments, only data from treatment-naive individuals were selected in this criterion. For criterion four (CRT≥325 µm), cases where data were missing were also included to prevent potential participants from being excluded.

In step 2, individuals meeting the specified criteria were identified based on their data within SMR. In cases of bilateral DMO, the eye with the poorest BCVA at diagnosis was included.

### Clinical parameters

Data from the original visit resulting in inclusion in SMR, (ie, the time when individuals were diagnosed with DMO) were compared with baseline characteristics of the study cohorts in YOSEMITE and RHINE. Variables available in both SMR and the phase III trials were incorporated into the analyses including age, gender, type 2 diabetes, BCVA in ETDRS if assessed and CRT. A similar comparison of these variables was also conducted between the treatment practice population and the phase III study cohorts.

### Statistical analysis

The selection process was carried out step by step for each criterion mentioned above to identify the proportion of eligible individuals in SMR meeting the YOSEMITE and RHINE main criteria. To evaluate similarities and disparities in demographic and clinical attributes between the cohorts from SMR and the trial cohorts, descriptive statistics were used. Student’s t-test was employed for continuous variables, while the χ^2^ test was used for categorical variables. Mean and SD were reported for continuous variables, whereas frequencies and proportions were reported for categorical variables. Sensitivity analyses were performed by omitting missing values for the CRT variable. The significance level for the p value was set at 5%. IBM SPSS Statistics V.28 and V.29 were used to conduct all analyses.

## Results

**Figure 1:**
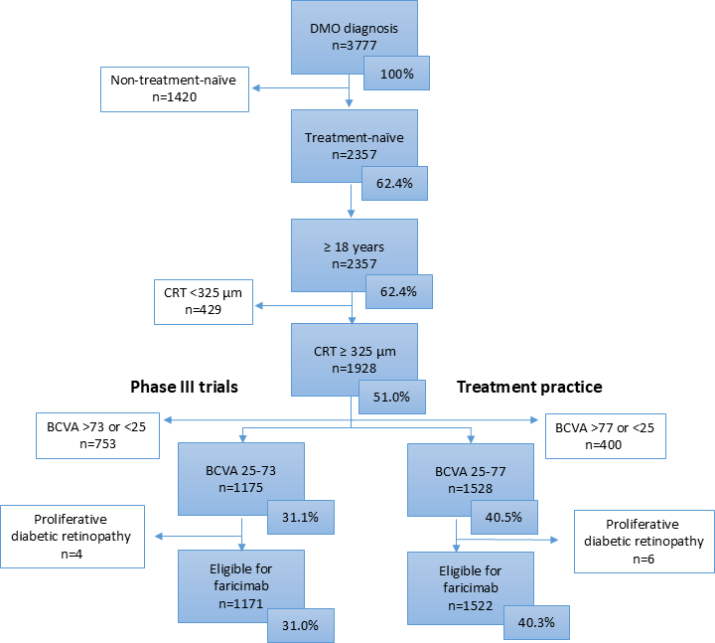
Selection of individuals in the Swedish Macula Registry (SMR) eligible for faricimab according to the main criteria in the YOSEMITE and RHINE phase III trials, as well as treatment practice. The exclusion criteria include registered proliferative retinopathy at the start of treatment. BCVA, best-corrected visual acuity; CRT, central retinal thickness; DMO, diabetic macular oedema.

In SMR, 3777 individuals were diagnosed with DMO between 1 January 2019 and 31 August 2023. The selection process based on the main entry criteria in the YOSEMITE and RHINE trials and treatment practice is shown in figure 1. Among the 3777 individuals with DMO, 2357 (62.4%) were treatment-naïve, all were 18 years or older, and 1928 (51.0%) had CRT≥325 µm. The BVCA criterion differed between the phase III trials and the treatment practice, where 1175 (31.1%) had 25–73 letters and 1528 (40.5%) had 25–77 letters, respectively. After excluding individuals with registration of proliferative disease at the start of treatment, a total of 1171 individuals (31.0%) met the criteria based on the main criteria of the phase III trials, while 1522 individuals (40.3%) met all criteria based on treatment practice. If only treatment-naïve individuals were considered, the eligible proportions correspond to 50% (1171 out of 2357) and 65% (1522 out of 2357), respectively, and the BCVA and CRT criteria were the most common reasons for non-eligibility.

Table 1 compares the eligible SMR population (n=1171) with the study populations in YOSEMITE and RHINE, respectively. The table reveals that the mean age in the SMR population was significantly higher compared with both YOSEMITE (67.5±11.6 vs 62.8±10.0, p<0.001) and RHINE (67.5±11.6 vs 61.6±10.1, p<0.001). The proportion of individuals with type 2 diabetes was significantly lower in the SMR compared with both YOSEMITE (85% vs 96%, p<0.001) and RHINE (85% vs 94%, p<0.001). BCVA was significantly lower in SMR compared with YOSEMITE (60±10 vs 62±10 letters, p<0.003) and RHINE (60±10 vs 63±9 letters, p<0.001). The CRT variable was significantly lower in SMR compared with both YOSEMITE (446±96 µm vs 486±131 µm, p<0.001) and RHINE (446±96 µm vs 471±127 µm, p=0.001). No significant difference was observed in terms of sex distribution.

**Table 1:** Characteristics for the eligible SMR population compared with the YOSEMITE and RHINE populations

	SMR(n=1171)	YOSEMITEfaricimab(n=313)	P value*	RHINEfaricimab(n=319)	P value†
Age (years), mean (SD)	67.5 (11.6)	62.8 (10.0)	<0.001	61.6 (10.1)	<0.001
Sex, n (%)			0.964		0.819
Female	430 (36.7%)	116 (37%)		120 (38%)	
Male	741 (63.3%)	197 (63%)		199 (62%)	
Type 2 diabetes, n (%)	990 (84.5%)	299 (96%)	<0.001	300 (94%)	<0.001
BCVA (ETDRS letters), mean (SD)	60 (10.2)	62 (10.2)	<0.003	63 (9.3)	<0.001
CRT (μm), n (SD)	446 (101.3)	486 (130.8)	<0.001	471 (127.0)	0.001

YOSEMITE and RHINE, phase III studies investigating the efficacy, duration and safety of faricimab compared to aflibercept in DMO.

* pP- value refers to SMR compared tocompared with YOSEMITE.

† P value refers to SMR compared with RHINE.

BCVA, best-corrected visual acuity; CRT, central retinal thickness; DMO, diabetic macular oedema; ETDRSEarly Treatment Diabetic Retinopathy StudySMR, Swedish Macula Register

Table 2 compares the eligible treatment practice population (n=1522) with the YOSEMITE and RHINE populations, respectively. The table presents findings consistent with those of the SMR population, demonstrating that the mean age was significantly higher, the prevalence of type 2 diabetes was lower, the CRT was lower and no difference in sex distribution in the treatment practice population compared with both the YOSEMITE and RHINE populations. Similarly, BCVA was significantly higher in the treatment practice population compared with YOSEMITE (64±11 vs 62±10 letters, p=0.013). However, no significant difference was observed in BCVA between the treatment practice population and RHINE (64±11 vs 63±9 letters, p=0.092).

**Table 2:** Characteristics for the eligible treatment practice population compared with the YOSEMITE and RHINE populations

	SMR(n=1522)	YOSEMITEfaricimab(n=313)	P value*	RHINEfaricimab(n=319)	P value†
Age (years), mean (SD)	66.8 (12.1)	62.8 (10.0)	<0.001	61.6 (10.1)	<0.001
Sex, n (%)			0.824		0.679
Female	551 (36.2%)	116 (37%)		120 (38%)	
Male	971 (63.8%)	197 (63%)		199 (62%)	
Type 2 diabetes, n (%)	1274 (83.7%)	299 (96%)	<0.001	300 (94%)	<0.001
BCVA (ETDRS letters), mean (SD)	64 (11.0)	62 (10.2)	0.013	63 (9.3)	0.092
CRT (μm), n (SD)	437 (96.2)	486 (130.8)	<0.001	471 (127.0)	<0.001

YOSEMITE and RHINE, phase III studies investigating the efficacy, duration and safety of faricimab compared to aflibercept in DMO.

* pP- value refers to SMR compared tocompared with YOSEMITE.

† P value refers to SMR compared with RHINE.

BCVA, best-corrected visual acuity; CRT, central retinal thickness; DMO, diabetic macular oedema; ETDRSEarly Treatment Diabetic Retinopathy StudySMR, Swedish Macula Register

Sensitivity analyses conducted to investigate the impact of missing data of the CRT variable revealed that 652 (17.3%) individuals in the SMR met all the criteria for faricimab based on YOSEMITE and RHINE main criteria (online supplemental figure 1). Meanwhile, 821 (21.7%) individuals met all the criteria for faricimab based on criteria from treatment practice. Among treatment-naïve individuals, this corresponds to 28% (652 out of 2357) and 35% (821 out of 2357) eligibility in the SMR and treatment practice cohorts, respectively. The characteristics of the eligible SMR and treatment practice cohorts showed similar differences to the YOSEMITE and RHINE populations as observed in the primary analyses (online supplemental tables 1 and 2).

## Discussion

The study revealed that barely a third of the individuals with DMO in the SMR dataset met the main criteria from the YOSEMITE and RHINE trials for faricimab. This contrasts with 40% of individuals meeting the criteria for faricimab based on treatment practice national guidelines. The largest drop-off in individuals occurred during the selection of treatment-naive patients. This can be attributed to many individuals in the SMR database having received DMO treatment for an extended period, thus not receiving their diagnosis within the last 5 years. Among treatment-naïve individuals, the largest drop-off in individuals occurred due to the BCVA criterion, followed by CRT.

A higher proportion of individuals met the treatment practice criteria (40%) compared with the clinical trial criteria (31%). This was expected as guidelines in treatment practice had wider intervals for visual acuity, resulting in more individuals meeting the criterion. The extended treatment intervals in clinical practice are attributed to treatment initiation at higher visual acuity levels as per national guidelines, coupled with a screening programme for early detection of sight-threatening retinopathy in individuals with diabetes, where good visual acuity persists even at early disease stages. Moreover, the proportion of eligible individuals in SMR (31%) is lower than had been found in a similar DMO study. Stewart *et al*^17^ conducted a retrospective single-centre study to evaluate the eligibility of ranibizumab among 216 eyes of 176 individuals, identified 55%–63% that met the criteria from three landmark randomised controlled trials. About one-third did not meet the eligibility criteria, mainly because of too low or too high BCVA, followed by recently received different DMO treatments, hence not being treatment naïve. Even though the applied eligibility criteria were not the same as the current study, the patient demographics (mean age, sex, diabetes type and baseline BCVA) and the most common reasons for exclusion were in accordance. Comparing the eligibility among treatment-naïve individuals in the current study, the proportions are more evenly aligned. In contrast, the eligible proportion aligns closely with findings from our prior study by Norberg *et al*^12^ where a real-world heart failure cohort (n=2029) was assessed for sacubitril-valsartan eligibility. Results revealed that only 24% of the single-centre heart failure population met the main criteria of the PARADIGM-HF trial, but this figure increased to 40% when more pragmatic criteria resembling clinical practice were applied. Both this study and our current investigation suggest that broader eligibility criteria result in a larger pool of eligible individuals for new treatments. Expanding the criteria in clinical trials would make the evidence better reflect real-world populations, thereby improving the generalisability of results to clinical practice.

The most notable distinctions between the SMR/treatment practice cohorts and the YOSEMITE/RHINE populations were age and CRT. Regarding age, individuals in the SMR and treatment practice cohorts were significantly 4–5.9 years older compared with the trial populations. Despite the higher age, they had a lower CRT, indicating slightly healthier SMR populations compared with trial populations, which could be due to the national screening programme which enables early diagnosis and identifies if the diabetic treatment needs to be optimised. Conversely, the mean BCVA values were relatively similar between all cohorts, which may be due to the visual acuity being affected by various factors common among older people, such as age degeneration or cataracts.

The key strengths of this study lie in the utilisation of a national registry to investigate the purpose and address the research questions. By using SMR, access to large volumes of data was facilitated due to its national coverage. Additionally, the registry’s content is based on real patient cases, and the participation rate of clinics was high at 80% in 2023, enhancing the generalisability of the study to the Swedish population as the analyses are grounded in real clinical scenarios. Furthermore, selection bias is mitigated through the usage of a national registry, as the individuals included represent nearly all individuals receiving anti-VEGF treatment in Sweden, thus constituting a more representative population compared with clinical trials that involve selected cohorts. Regarding the seven-step systematic implementation approach, it proved to be useful and efficient in identifying individuals eligible for faricimab in DMO. By applying the systematic approach, a more precise estimation of the number of suitable individuals who may be eligible for a new treatment ahead of implementation can be obtained. Consequently, this can facilitate healthcare prioritisation for patients who may benefit from the new treatment and enable better budget planning for the clinics.

This study is subject to several limitations. First, is the presence of missing data in SMR, particularly concerning the CRT variable. To evaluate the impact of this missing data, a sensitivity analysis was performed. The analysis revealed that the absence of data for the CRT variable did not significantly alter the conclusions. This finding suggests that the comparison of variables and the generalisability of the results were largely unaffected. Second, there is a risk of inaccurate data entry in quality registries, and a prior study has shown that SMR contains 5% erroneous registrations for ETDRS and up to 13% for other variables.^18^ To address this, SMR has implemented a background validation, which entails the existence of a reference interval to avoid registration of unreasonable values and fixed options for certain variables.^11^ Some variables are also made mandatory to reduce the risk of important variables being forgotten or incorrectly filled in. Third, the SMR lacked certain inclusion and exclusion criteria from the YOSEMITE and RHINE trials, leading to the utilisation of only available variables in the study. This may affect the comparison of populations and, consequently, the transferability of the results to clinical practice. Fourth, this study only included treatment-naive individuals, whereas the phase III trials, YOSEMITE and RHINE, included up to 20%–22% previously treated individuals, provided that 3 months had elapsed since treatment initiation and the start of the studies. This difference may impact the study’s results because treatment-naive and non-treatment-naive individuals may exhibit differences in disease severity and outcomes, such as visual acuity. As mentioned earlier, the largest drop-off of individuals occurred when selecting treatment-naive individuals. It is possible that the dropout rate would have been reduced at this criterion if both treatment-naive and previously treated individuals had been included in the same manner as in the YOSEMITE and RHINE trials. This may affect the generalisability to the broader population and should therefore be considered when interpreting the results. Fifth, in 2020 the COVID-19 pandemic in Sweden led to fewer appointments and treatments for eye conditions like DMO and nAMD, as some people delayed or cancelled their visits.^11^ Treatments gradually returned to normal levels after June 2020. Despite the decrease in visits, individuals experiencing visual impairment due to missed treatment later rescheduled appointments for treatment. This prioritisation of active treatment over visits and examinations during the COVID-19 period may have impacted the outcome, particularly visual acuity, in SMR.

Faricimab is a novel drug, with limited clinical experience in real-world settings regarding long-term effects and safety concerns. Further information from the ongoing clinical trials, RHONE-X, VOYAGER and AVONELLE-X, will provide insights into the extended outcomes for patients receiving faricimab treatment, as well as those transitioning from aflibercept to faricimab after completing the phase III trials.^19^ So far, real-world studies have shown that faricimab has been tolerated similarly as in the clinical trials and the effectiveness seems to persist also in more heterogenous real-world populations. Prolonged treatment intervals are considered to reduce the burden on individuals and benefit healthcare in terms of resources and costs.^20^ As faricimab emerges as a promising therapeutic avenue, its prospective role in shaping the landscape of DMO treatment warrants vigilant observation and further investigation.

## Conclusion

Nearly one-third of individuals with DMO in the SMR met the YOSEMITE and RHINE main trial criteria, contrasting with 40% based on Swedish treatment practice. The SMR and treatment practice cohorts exhibited slightly advanced age and less severe DMO compared with the trial cohorts. Further research into the safety profile of faricimab in clinical settings is necessary, along with the consideration of additional eligibility criteria when implementing faricimab in ophthalmology care.

## supplementary material

10.1136/bmjopen-2024-089801 online supplemental file 1

## Data Availability

Data are available upon reasonable request.
