# Supplementary material for: Eligibility for faricimab in a real-world diabetic macular oedema population: a cross-sectional study
Source: BMJ Open. 2025 Feb 5;15(2):e089801. doi: 10.1136/bmjopen-2024-089801 (PMC11800199; doi:10.1136/bmjopen-2024-089801)
Supplement: online supplemental file 1 [file bmjopen-15-2-s001.pdf]

Supplemental material

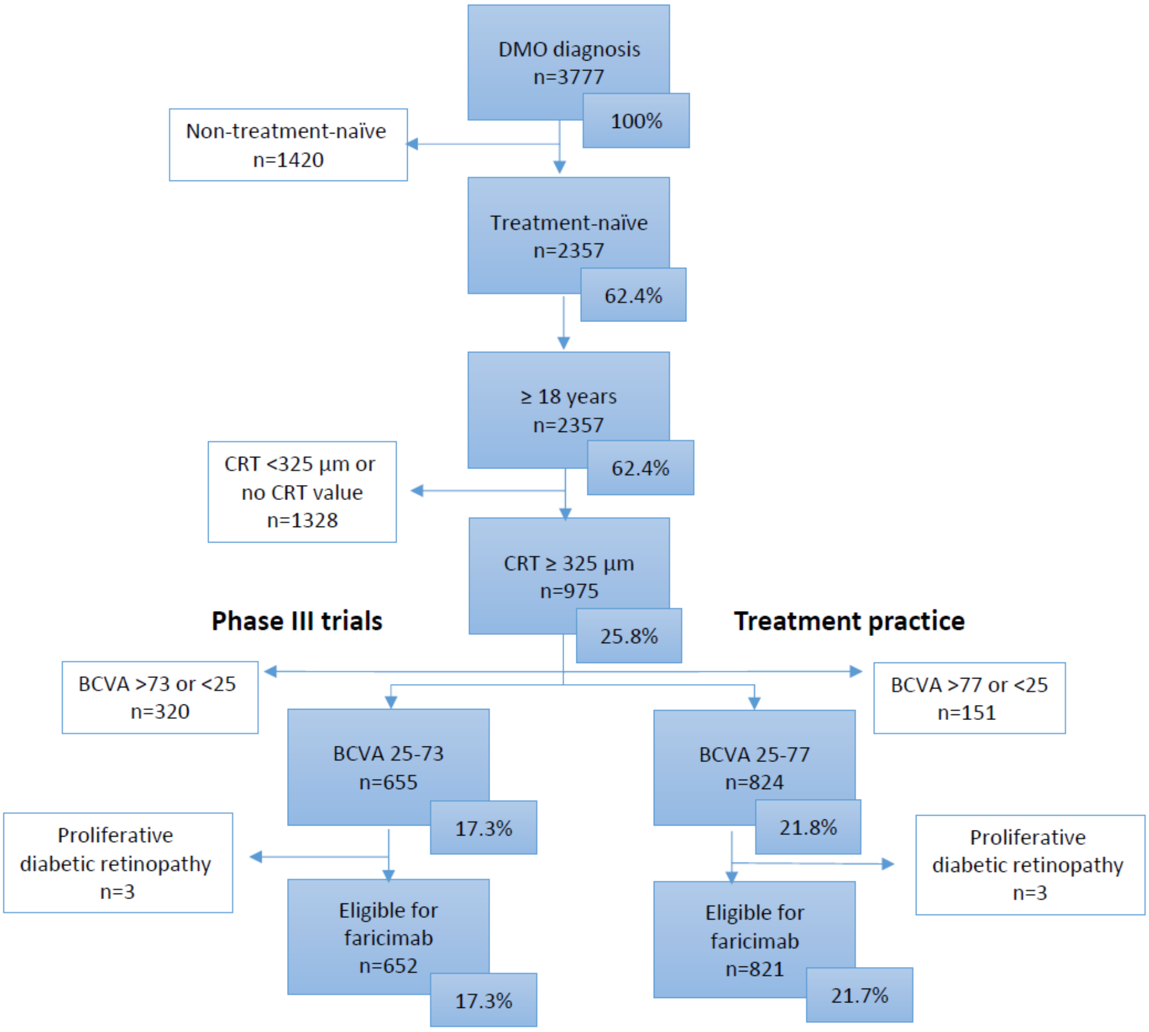

Supplementary Figure 1. Sensitivity analyses excluding missing central retinal thickness (CRT) values for the selection of individuals in the Swedish Macula Registry (SMR) eligible for faricimab treatment, based on the main criteria from the YOSEMITE and RHINE phase III trials, as well as treatment practice. BCVA, best-corrected visual acuity; DMO, diabetic macular oedema; Exclusion criteria: registered proliferative retinopathy at start of treatment.

.

Supplementary Table 1. Sensitivity analyses excluding missing central retinal thickness (CRT) values for the eligible Swedish Macula Registry (SMR) population compared to the YOSEMITE and RHINE phase III trial populations.

	SMR (n = 652)	YOSEMITE faricimab (n = 313)	P value*	RHINE faricimab (n = 319)	P value ǂ
**Age (years), mean (SD)**	66.7 (11.9)	62.8 (10.0)	<0.001	61.6 (10.1)	<0.001
**Sex, n (%)**			1.000		0.935
Female	242 (37.1 %)	116 (37 %)		120 (38 %)	
Male	410 (62.9 %)	197 (63 %)		199 (62 %)	
**Type 2 diabetes, n (%)**	573 (87.9 %)	299 (96 %)	<0.001	300 (94 %)	0.004
**BCVA (ETDRS letters), mean (SD)**	60 (10,4)	62 (10.2)	0.002	63 (9.3)	<0.001
**CRT (µm), n (SD)**	447 (99.8)	486 (130.8)	<0.001	471 (127.0)	0.003

YOSEMITE and RHINE, phase III studies investigating the efficacy, duration, and safety of faricimab compared to aflibercept in DMO.

*P value refers to SMR compared to YOSEMITE.

ǂP value refers to SMR compared to RHINE.

BCVA, best-corrected visual acuity; CRT, central retinal thickness; DMO, diabetic macular oedema; ETDRS = early treatment diabetic retinopathy study; SD, standard deviation; SMR, Swedish macula register; YOSEMITE and RHINE, phase III studies investigating the efficacy, duration, and safety of faricimab compared to aflibercept in DMO.

Supplementary Table 2. Sensitivity analyses excluding missing central retinal thickness (CRT) values for the eligible Treatment practice population compared to the YOSEMITE and RHINE phase III trial populations.

	SMR (n = 821)	YOSEMITE faricimab (n = 313)	P value*	RHINE faricimab (n = 319)	P value ǂ
**Age (years), mean (SD)**	66.5 (11.9)	62.8 (10.0)	<0.001	61.6 (10.1)	<0.001
**Sex, n (%)**			0.661		0.537
Female	291 (35.4 %)	116 (37 %)		120 (38 %)	
Male	530 64.6 %)	197 (63 %)		199 (62 %)	
**Type 2 diabetes, n (%)**	715 (87.1 %)	299 (96 %)	<0.001	300 (94 %)	0,001
**BCVA (ETDRS letters), mean (SD)**	63 (11.2)	62 (10,2)	0.152	63 (9.3)	0.539
**CRT (µm), n (SD)**	438 (95.1)	486 (130.8)	<0.001	471 (127.0)	<0.001

YOSEMITE and RHINE, phase III studies investigating the efficacy, duration, and safety of faricimab compared to aflibercept in DMO.

*P value refers to SMR compared to YOSEMITE.

ǂP value refers to SMR compared to RHINE.

BCVA, best-corrected visual acuity; CRT, central retinal thickness; DMO, diabetic macular edema; ETDRS = early treatment diabetic retinopathy study; SD, standard deviation; SMR, Swedish macula register; YOSEMITE and RHINE, phase III studies investigating the efficacy, duration, and safety of faricimab compared to aflibercept in DMO.
