# Supplementary material for: Eligibility for faricimab in a real-world diabetic macular oedema population: a cross-sectional study
Source: BMJ Open. 2025 Feb 5;15(2):e089801. doi: 10.1136/bmjopen-2024-089801 (PMC11800199; doi:10.1136/bmjopen-2024-089801)
Supplement: Supplementary file 2 [file bmjopen-2024-089801.reviewer_comments.pdf]

PEER REVIEW HISTORY

BMJ Open publishes all reviews undertaken for accepted manuscripts. Reviewers are asked to complete a checklist review form and are provided with free text boxes to elaborate on their assessment. These free text comments are reproduced below.

ARTICLE DETAILS

Title (Provisional)

Eligibility for faricimab in a real-world diabetic macular edema population: a cross-sectional study

Authors

Westborg, Inger; Al-Najjar, Ayat; Norberg, Helena

VERSION 1 - REVIEW

**Reviewer**	**1**
**Name**	**Amoaku, W.**
**Affiliation**	**Univ Nottingham, Ophthalmology**
**Date**	**19-Aug-2024**
**COI**	**None**

This manuscript requires a few minor clarifications and edits.

Clarity is required in the 2 cohorts. How do the SMR and the treatment practice populations differ?

What do the authors define as 'proliferative DME'? Do they mean proliferative DR? This needs attention in lines 26,33, 133 and elsewhere.

What do they consider high risk proliferative disease (under exclusions)?

Line 89: 'county' or 'country'?

Line 260: spelling 'fourth'

Line 225: 'eye-c'?

**Reviewer**	**2**
**Name**	**Liberski , Slawomir**
**Affiliation**	**Poznan University of Medical Sciences, Ophthalmology**
**Date**	**13-Oct-2024**

COI **none**

I read with great interest the article that investigates the eligibility for faricimab in a real-world diabetic macular edema (DME) population to one of the most important clinical trials in ophthalmology - RHINE and YOSEMITE and to compare the eligible DME populations to this trial populations. First, I want to highlight the relevance of the topic addressed in the article. Good quality articles are of great value in supporting the management of nAMD patients in this context.

Having said that, I would like to make a few comments:

1) Introduction - apparently introduces the reader to the manuscript and the issues studied - I have no comments on this section

2) Materials and methods - this section is extensive and extensively introduces the reader to the assumptions and methods of the study

Line 125: Please extend information about eye-c

3) Results - the results of the study are transparently presented

Line 166: Please insert the correct (English) form of the month's name

4) Discussion—This section comprehensively discusses the study's results, including the differences between the SMR population and the populations studied in the RHINE and YOSEMITE trials. I suggest replacing the study's strengths and limitations at the end of the discussion section. However, this is only a stylistic issue.

In conclusion, this is a very valuable manuscript; I read it with great interest and recommend its acceptance after minor revisions.

Reviewer **3**

Name **Chung, Yoo-Ri**

**Affiliation**	**Ajou University School of Medicine**
**Date**	**22-Nov-2024**
**COI**	**I have no competing interests.**

Authors in the manuscript titled "Eligibility for faricimab in a real-world diabetic macular edema population: a cross-sectional study" have investigated the difference between phase 3 controlled trials and real-world populations for diabetic macular edema (DME) patients. The authors used the Swedish Macula Registry (SMR) as a primary group and 'treatment practice' group as a secondary group, and compared them to the YOSEMITE and RHINE trials.

In real-world clinical practice, clinicians often try intravitreal injections in DME patients with lower or higher visual acuity, i.e. out of the 25-73 letters. This tendency can be noted in DME eyes with focal thickening that may not fulfill the CRT of 325 um or more. The only different eligibility criterion was that of vision defined by the authors as 25-77 letters in the treatment practice group, which difference is not significant from 25-73 letters. For me, the application of the secondary election group does not add any specific significance.

As the authors mentioned, treatment-naïve DME patients were approximately 20% in YOSEMITE and RHINE trials while 30% of SMR fulfilled the same criteria. According to the authors, this reaches nearly 40% by following the national Swedish guidelines. However, in sensitivity analyses that excluded those without CRT data reached 21.7% in treatment practice group, which results was similar to the clinical trials. This does not correlate with the conclusions of the authors.

VERSION 1 - AUTHOR RESPONSE

Reviewer: 1
Dr. W. Amoaku, Univ Nottingham
Comments to the Author:
This manuscript requires a few minor clarifications and edits.
Clarity is required in the 2 cohorts. How do the SMR and the treatment practice populations differ?

Answer: Thank you for your comment. The only different eligibility criterion was that of vision defined as 25-73 letters in the SMR population (based on YOSEMITE/RHINE trials) and 25-77 letters in the treatment practice group (based on national guidelines in Sweden). We know that the difference in number of letters is small, but the extended visual acuity means that a larger proportion of patients are eligible for treatment (40,3% vs 31.0%). National guidelines might therefore be of importance for treatment initiation. Hence, we wanted to study this population in addition to the phase 3 trial criteria.

To clarify this in the paper we have added the following to the Methods section in page 6 (lines 15-20):

“Additionally, a secondary screening of eligible individuals was performed using the same criteria, except for BCVA, which ranged from 25 to 77 ETDRS letters. This was due to the national guidelines and we wanted to study the results from this perspective as it could have implications on initiation and treatment practice in Sweden.[16]”

What do the authors define as 'proliferative DME'? Do they mean proliferative DR? This needs attention in lines 26,33, 133 and elsewhere.

Answer: Thank you, corrections are made to proliferative diabetic retinopathy both in the paper and Figures.

What do they consider high risk proliferative disease (under exclusions)?

Answer: Proliferative diabetic retinopathy can be registered in SMR as an indication for treatment and all patients treated for proliferative disease were excluded. There is also possible to record diabetes complications as proliferations at start of treatment or during course of treatment. There were a few patients registered for DME treatment that also had registered complications as proliferative disease at start of treatment. These patients were excluded as it could be an incorrect registration of indication. We have corrected the exclusion criteria.

Line 89: 'county' or 'country'?

Answer: Thank you. We realise that this can be misinterpreted and have changed this to regional hospital instead.

Line 260: spelling 'fourth'
Answer: Thank you, the misspelling has been corrected.

Line 225: 'eye-c'?

Answer: We apologise for the inadvertent spacing error in the text, the correct term is ophthalmologist.

Reviewer: 2
Dr. Slawomir Liberski , Poznan University of Medical Sciences
Comments to the Author:
I read with great interest the article that investigates the eligibility for faricimab in a real-world diabetic macular edema (DME) population to one of the most important clinical trials in ophthalmology - RHINE and YOSEMITE and to compare the eligible DME populations to this trial populations. First, I want to highlight the relevance of the topic addressed in the article. Good quality articles are of great value in supporting the management of nAMD patients in this context.

Having said that, I would like to make a few comments:

1) Introduction - apparently introduces the reader to the manuscript and the issues studied - I have no comments on this section

2) Materials and methods - this section is extensive and extensively introduces the reader to the assumptions and methods of the study

Line 125: Please extend information about eye-c

Answer: We apologise for the inadvertent spacing error in the text, the correct term is ophthalmologist.

3) Results - the results of the study are transparently presented

Line 166: Please insert the correct (English) form of the month's name

Answer: Thank you, the correction has been made.

4) Discussion—This section comprehensively discusses the study's results, including the differences between the SMR population and the populations studied in the RHINE and YOSEMITE trials. I suggest replacing the study's strengths and limitations at the end of the discussion section. However, this is only a stylistic issue.

Answer: Thank you for the suggestion, we have moved the strengths and limitations to the end of the Discussion section.

In conclusion, this is a very valuable manuscript; I read it with great interest and recommend its acceptance after minor revisions.

Answer: Thank you for your wise comments.

Reviewer: 3
Dr. Yoo-Ri Chung, Ajou University School of Medicine
Comments to the Author:
Authors in the manuscript titled "Eligibility for faricimab in a real-world diabetic macular edema population: a cross-sectional study" have investigated the difference between phase 3 controlled trials and real-world populations for diabetic macular edema (DME) patients. The authors used the Swedish Macula Registry (SMR) as a primary group and 'treatment practice' group as a secondary group, and compared them to the YOSEMITE and RHINE trials.

In real-world clinical practice, clinicians often try intravitreal injections in DME patients with lower or higher visual acuity, i.e. out of the 25-73 letters. This tendency can be noted in DME eyes with focal thickening that may not fulfill the CRT of 325 um or more. The only different eligibility criterion was that of vision defined by the authors as 25-77 letters in the treatment practice group, which difference is not significant from 25-73 letters. For me, the application of the secondary election group does not add any specific significance.

Answer: Thank you for your comment and we agree that the difference in number of letters is small, but the extended visual acuity means that a larger proportion of patients are eligible for treatment (40.3% vs 31.0%). National guidelines might therefore be of importance for treatment

initiation. We want to show that small changes in inclusion criteria can have an impact on the number of eligible patients.

We have added the following to the Methods section in page 6 (lines 15-20):

"Additionally, a secondary screening of eligible individuals was performed using the same criteria, except for BCVA, which ranged from 25 to 77 ETDRS letters. This is due to the national guidelines and we wanted to study the results from this perspective as it could have implications on initiation and treatment practice in Sweden.[16]"

As the authors mentioned, treatment-naïve DME patients were approximately 20% in YOSEMITE and RHINE trials while 30% of SMR fulfilled the same criteria. According to the authors, this reaches nearly 40% by following the national Swedish guidelines. However, in sensitivity analyses that excluded those without CRT data reached 21.7% in treatment practice group, which results was similar to the clinical trials. This does not correlate with the conclusions of the authors.

Answer: Thank you for your comment. We realize that these numbers with treatment-naïve individuals are possible to misinterpret. We have therefore made several revisions.

First, we would like to explain that in our SMR population we could only include treatment-naïve individuals. We have tried to explain this in the Methods section and have now added the yellow text below (page 6, lines 22-30):

"Regarding criterion two, YOSEMITE and RHINE stipulated that individuals should either be treatment-naïve or have received treatment previously, provided that the treatment occurred no later than three months before the start of the study.[9] Hence, YOSEMITE and RHINE allowed for 22% and 20% non-treatment-naïve individuals. Because SMR lacks information regarding the timing of prior treatments, only data from treatment-naive individuals were selected in this criterion."

And further addressed this in the limitations in the Discussion section (page 11, from line 48):

"Fourth, this study only included treatment-naive individuals, whereas the phase III trials, YOSEMITE and RHINE, included up to 20-22% previously treated individuals, provided that three months had elapsed since treatment initiation and the start of the studies. This difference may impact the study's results because treatment-naive and non-treatment-naive individuals may exhibit differences in disease severity and outcomes, such as visual acuity. As mentioned earlier, the largest drop-off of individuals occurred when selecting treatment-naive individuals. It is possible that the dropout rate would have been reduced at this criterion if both treatment-naive and previously treated individuals had been included in the same manner as in the YOSEMITE and RHINE trials. This may affect the generalizability to the broader population and should therefore be considered when interpreting the results."

Furthermore, we have omitted the sentences regarding treatment-naïve individuals from the abstract and conclusions because this is not our primary results.

To clarify the treatment-naïve numbers we have added the following to the Results section, page 7 (lines 28-32):

"If only treatment-naïve individuals were considered, the eligible proportions correspond to 50% (1171 out of 2357) and 65% (1522 out of 2357), respectively, and the BCVA and CRT criteria were the most common reasons for non-eligibility.

Also, regarding the Sensitivity analysis (concerning the impact of missing data of the CRT variable) in the Results section page 9 (lines 40-43) we have added similar information. It can be noted that these proportions in the sensitivity analysis are similar to the eligible proportions in the main analysis:

"Among treatment-naïve individuals, this corresponds to 28% (652 out of 2357) and 35% (821 out of 2357) eligibility in the SMR and treatment practice cohorts, respectively."

We hope that our revisions are satisfactory, and we are open to further revisions if needed.

VERSION 2 - REVIEW

**Reviewer**	**2**
**Name**	**Liberski , Slawomir**
**Affiliation**	**Poznan University of Medical Sciences, Ophthalmology**
**Date**	**12-Jan-2025**
**COI**	

The authors provided satisfactory responses to all my comments and suggestions. I have no further comments on this work. I recommend its acceptance.
